# Comparative Pro-cognitive and Neurochemical Profiles of Glycine Modulatory Site Agonists and Glycine Reuptake Inhibitors in the Rat: Potential Relevance to Cognitive Dysfunction and Its Management

**DOI:** 10.1007/s12035-020-01875-9

**Published:** 2020-01-20

**Authors:** Kevin C.F. Fone, David J.G. Watson, Rodolphe I. Billiras, Dorothee I. Sicard, Anne Dekeyne, Jean-Michel Rivet, Alain Gobert, Mark J. Millan

**Affiliations:** 1 School of Life Sciences, Queen’s Medical Centre, The University of Nottingham, Nottingham, NG7 2UH UK; 2 Centre for Therapeutic Innovation-CNS, Institut de Recherche Servier, Centre de Recherches de Croissy, 125 Chemin de Ronde, 78290 Croissy-sur-Seine, France

**Keywords:** NMDA receptor, Glycine modulatory site, D-serine, Cognition, Social cognition, Prefrontal cortex, Schizophrenia, Microdialysis

## Abstract

Frontocortical NMDA receptors are pivotal in regulating cognition and mood, are hypofunctional in schizophrenia, and may contribute to autistic spectrum disorders. Despite extensive interest in agents potentiating activity at the co-agonist glycine modulatory site, few comparative functional studies exist. This study systematically compared the actions of the glycine reuptake inhibitors, sarcosine (40–200 mg/kg) and ORG24598 (0.63–5 mg/kg), the agonists, glycine (40–800 mg/kg), and D-serine (10–160 mg/kg) and the partial agonists, S18841 (2.5 mg/kg s.c.) and D-cycloserine (2.5–40 mg/kg) that all dose-dependently prevented scopolamine disruption of social recognition in adult rats. Over similar dose ranges, they also prevented a delay-induced impairment of novel object recognition (NOR). Glycine reuptake inhibitors specifically elevated glycine but not D-serine levels in rat prefrontal cortical (PFC) microdialysates, while glycine and D-serine markedly increased levels of glycine and D-serine, respectively. D-Cycloserine slightly elevated D-serine levels. Conversely, S18841 exerted no influence on glycine, D-serine, other amino acids, monamines, or acetylcholine. Reversal of NOR deficits by systemic S18841 was prevented by the NMDA receptor antagonist, CPP (20 mg/kg), and the glycine modulatory site antagonist, L701,324 (10 mg/kg). S18841 blocked deficits in NOR following microinjection into the PFC (2.5–10 μg/side) but not the striatum. Finally, in rats socially isolated from weaning (a neurodevelopmental model of schizophrenia), S18841 (2.5 and 10 mg/kg s.c.) reversed impairment of NOR and contextual fear-motivated learning without altering isolation-induced hyperactivity. In conclusion, despite contrasting neurochemical profiles, partial glycine site agonists and glycine reuptake inhibitors exhibit comparable pro-cognitive effects in rats of potential relevance to treatment of schizophrenia and other brain disorders where cognitive performance is impaired.

**Electronic supplementary material:**

The online version of this article (10.1007/s12035-020-01875-9) contains supplementary material, which is available to authorized users.

## Introduction

The role of glutamatergic dysfunction in psychiatric disorders is well established, and N-methyl-D-aspartate (NMDA) receptors retain special interest because of their broad cortical and subcortical distribution, localisation on GABAergic interneurons and pyramidal cells, and key role in regulation of neuroplasticity, cognition, epilepsy, mood and motor behaviour. Furthermore, the NMDA channel blocker, ketamine is attracting interest as a fast-acting antidepressant which is metabolised to (2S,6S;2R,6R)-hydroxynorketamine that activates AMPA receptors [1] and causes mTOR- and BDNF-dependent increased dendritic arborisation in dopaminergic neurons which may be beneficial to other mood disorders [2, 3]. Furthermore, agents *promoting* NMDA activity are also of potential use to treat Alzheimer’s disease, Parkinson’s disease, autism spectrum disorders (ASDs), and schizophrenia [4, 5].

Glutamate activation of the NMDA receptor requires concurrent binding of glycine or, predominantly in the PFC, D-serine at the glycine modulatory site [6–8]. Thus, pharmacological modulation of the glycine site has been prioritised to restore hypofunction by direct agonism, inhibition of enzymes regulating serine metabolism or increasing synaptic glycine by inhibition of glycine transporters [9–12]. Although glycine and D-serine have been extensively characterised, it has proven very hard to generate small molecule, positive modulators of the glycine site. Recently, the partial agonists GLYX-13 (rapastinel) [13] and ((2S, 3R)-3-hydroxy-2-((R)-5-isobutyryl-1-oxo-2,5-diazaspiro[3,4]octan-2-yl)butanamide) NYX-2925 [14] were shown to enhance hippocampal LTP and cognition in rodent paradigms. Although the cyclic imidazolinone, S18841 [15], was not developed clinically, this glycine site partial agonist is a very useful ligand for exploitation in parallel with the prototypical partial agonist D-cycloserine to identify the functional roles of the glycine modulatory site and ascertain underlying potentially beneficial therapeutic mechanisms. This approach was supported by the current findings that unlike other agents tested, S18841 did not significantly elevate microdialysate glycine levels making it a particularly informative agent to examine the impact of partial agonism at the glycine site on cognitive function.

NMDA receptor hypofunction is strongly implicated in the pathogenesis of schizophrenia [16, 17]. Moreover, increased levels of the endogenous NMDA receptor antagonists, N-acetylaspartylglutamate (NAAG), and kynurenate, occur in the cortex and hippocampus [18] along with decreased plasma D-serine [19] but see [20] and glycine in schizophrenia, and depletion of the latter correlates with severity of negative symptoms [21]. Genetic and functional studies suggest alterations in the rate-limiting enzyme for D-serine synthesis, D-serine racemase, and the major metabolising enzyme D-amino oxidase particularly in the prefrontal cortex (PFC) may contribute to NMDA receptor hypofunction in schizophrenia [16, 22]. D-Cycloserine and the tetrapeptide glycine partial agonist, GLIX-13, improve social behaviour in mice models of ASD [23] and D-cycloserine alleviated anxiety, social and vocal communication changes in the valproate rat model of ASD [24] as well as attenuating stereotype behaviour in 20 young ASD patients [25], but there have been few other clinical trials.

Over 70 placebo-controlled clinical trials with glycine modulatory site compounds (including glycine, D-serine, D-cycloserine and sodium benzoate) often given as adjuncts to antipsychotics have shown inconsistent and at best modest improvement in negative symptoms or cognitive domains [10, 17, 26]. Such inconsistent findings could be due to a bell-shaped dose response effect; desensitisation with chronic administration of glycine agonists, or muscarinic actions of antipsychotics preventing any cognitive improvement (as discussed fully later); or variation in the pharmacological effect of glycine agonists, partial agonists and GlyT1 inhibitors used, a possibility investigated herein. In comparison, another recent meta-analysis showed that NMDA modulators (glycine and D-serine) were more effective than amisulpiride, risperidone and olanzepine at reducing negative symptoms in adolescents at clinical high risk for developing psychosis [27]. Therefore, such drugs may be of potential use for preventing the transition of ultra-high-risk subjects to full-blown psychosis and schizophrenia [28, 29]. They also warrant further investigation as a treatment for ASD, particularly if preclinical evidence could identify unique pharmacological mechanisms associated with any benefit.

GlyT1 inhibitors like sarcosine, ORG25935, and bitopertin (RG1678), increase NMDA receptor-dependent hippocampal LTP [30] and attenuate PCP-induced hyper-responsiveness to d-amphetamine in rodent models [30]. A few studies have also shown the impact of GlyT1 inhibitors on behaviour in animal models for psychiatric disorders. The GlyT1 inhibitor, ALX-5407, increased prepulse inhibition of acoustic startle (PPI) in DAB/2 mice with naturally low PPI [31], ASP2535 reversed a neonatal phencyclidine-induced impairment of NOR [32], and ORG25935 reversed the attention deficit seen in the 5-choice continuous performance task in a transcription factor specificity protein 4 hypomorphic mouse model for bipolar disorder and schizophrenia [33]. Further, bitopertin showed initial promise against negative symptoms in phase II trials [34]. Although phase II/III clinical studies were disappointing [35, 36], recent add-on trials of the D-amino acid oxidase inhibitor, sodium benzoate with the GlyT1 inhibitor, sarcosine, improved cognitive and global functioning in patients with schizophrenia [12], so clinical evaluation of drugs that reinforce NMDA receptor activity remains of interest. The current study was therefore performed to determine the key cortical neurotransmitters involved in the cognitive effects of agents that modify glycine function. This should, in turn, help identify reasons underlying the poor predictive translational value of animal models for clinical trials in schizophrenia and help identify potential new therapeutic targets to overcome cognitive and social dysfunction in schizophrenia and other CNS disorders such as ASD.

As discussed above, few studies have compared the utility of glycine site direct agonists, partial agonists, and GlyT1 inhibitors, in relevant rodent models to clarify the potential clinical utility of these mechanisms and novel agents to treat schizophrenia. Accordingly, we compared the glycine modulatory site agonists (glycine, D-serine, D-cycloserine and S18841) and GlyT1 inhibitors (sarcosine and ORG25935) on microdialysis levels of glycine and D-serine and other neurotransmitters thought to regulate cognition in the prefrontal cortex (PFC) of rats [37]. These compounds were also compared in three cognitive tasks: social recognition, novel object recognition (NOR) and an associative conditioned freezing response (CFR) task (selected for translational relevance to schizophrenia from consensus reviews [38, 39], and whose predictive validity will be considered in the discussion). In addition, we employed a microinjection procedure to examine the role of the PFC in the action of S18841.

Finally, we examined the effect of S18841 in a neurodevelopmental model, rearing rats in social isolation from weaning, that produces behavioural and neurochemical changes resembling schizophrenia [40, 41] including reduced PFC volume [42, 43] and hippocampal expression of genes involved in glutamate, GABA and dopamine neurotransmission [44]. This model is associated with an impairment in NOR [44–47], CFR [45, 46, 48] and social interaction [49], making it ideal for the current investigation.

## Materials and Methods

### Animals

Male Lister hooded rats (Charles River UK) were used for the novel object recognition (NOR) and isolation studies, while male Wistar rats (Charles River L’Arbresle, France) were used for the social recognition and microdialysis studies. All rats were housed in conventional open top cages with food and water available ad libitum under 12 h light/dark cycle (lights on 07.00 h) and constant temperature 21 ± 2 °C and humidity 55 ± 10%. All behavioural studies were performed between 08.00 and 16.00 h, and following every test, the apparatus was thoroughly cleaned with 20% *v*/*v* ethanol and dried to remove odour cues. All studies were performed using a blind protocol, so that the observer was unaware of drug treatment. Experiments were conducted in accordance with the Animals (Scientific Procedures) Act, 1986 and ARRIVE guidelines with approval of University of Nottingham Local Ethical Committee (behaviour) or EU guidelines (microdialysis). All efforts were made to minimise animal suffering, and the number of animals used.

### Extracellular Amino Acids Levels by Microdialysis

Male Wistar rats (225–300 g) supplied by Charles River (L’Arbresle, France) were housed in groups (3–4) with free access to food and water. Rats were anaesthetised under chloral hydrate (400 mg/kg, i.p.) and a guide cannula implanted above the PFC at coordinates: AP + 2.2, L ± 0.6 and DV − 0.2. [50]. Five days later, a cuprophane CMA/11 probe (4 mm) was implanted and perfused at 1 μl/min with a Ringer solution (NaCl 147.2 mM; KCl 4 mM and CaCl_2_ 2.3 mM buffered with phosphate at pH, 7.3) for 150 min to obtain stable baseline levels, and samples taken every 20 min thereafter for 3 h. Amino acid levels were measured in 5 μl dialysate using fluorimetric detection (ex 420 nm; em: 490 nm, FP2020plus, Jasco, Bouguenais, France) after separation by linear gradient chromatography (0% B to 40% B over 40 min where the mobile phase was ammonium acetate (50 mM, pH = 6.8, plus tetrahydrofurane (3%), and B was ammonium acetate (50 mM, pH = 6.8) plus acetonitrile (60%)) using a reverse phase column (Kromasil C18, 250 mm × 2.1 mm, particle size, 5 μm, Cluzeau Info Labo, Courbevoie, France) maintained at 44 °C and a flow rate of 0.4 ml/min as detailed elsewhere [51]. At the end of the experiment, sky blue was perfused through the probe and rats killed by pentobarbital overdose to verify correct probe placement by histology in 50 μm sections. Amino acid levels in fractional samples were expressed as a percentage of the mean value during 90 min baseline measurement immediately before treatment.

### Social Recognition

Social recognition, a cortical-dependent working memory task based on olfactory discrimination of a novel from a familiar juvenile rat, considered relevant to dysfunction seen in psychiatric disorders [52, 53], was examined in adult rats. The procedure, described elsewhere [54, 55], evaluated the promnesic actions of compounds on a scopolamine-induced (1.25 mg/kg s.c.) deficit. Adult male Wistar rats were individually housed for 2 days before testing. On the test day, a juvenile was placed into the home cage for a 5 min session. A second 5 min session was performed immediately after the first one with the same juvenile reintroduced (for evaluation of the promnesic actions of ligands on a scopolamine-induced (1.25 mg/kg s.c.) deficit of recognition). The time (s) spent in active social investigation (i.e., the time spent by adult rat in sniffing, following, biting, jumping and crawling over or under the juvenile) during the first (T1) and the second (T2) session was monitored. The difference “T2–T1” was calculated and dose-response curves produced.

### Social Isolation

The social isolation paradigm extensively validated in our laboratory [44–47, 56] produces a robust, reproducible behavioural syndrome including phenotype changes relevant to neurodevelopmental disorders with deficits in social behaviour and cognition such as schizophrenia, many of which are reversed by treatment with existing antipsychotics [46, 49]. The ability of acute injection of S18841 to reverse deficits in motor and cognitive behaviour was examined in this neurodevelopmental model. Rats were weaned at postnatal day (PND) 24, and half of each litter were housed either in social groups (3–4 per cage, 50 × 33 × 26 cm) or alone (41 × 24 × 20 cm) for the rest of the study. Isolated rats were housed in the same holding room having auditory and olfactory contact but no physical interaction with conspecifics. Rats were isolated for 5 weeks before undergoing a battery of behavioural tasks, arranged in order of least to most aversive (to minimise any sequential effect on subsequent tasks) at approximately 1-week intervals.

### Locomotor Activity

Locomotor activity (ambulation and rearing) was measured using infrared beams during a 1 h period as described before and in detail in the supplementary information [49, 57].

### Novel Object Recognition

NOR is a visual learning and memory task that relies on innate preference of rats for novelty. It does not require previous training or re-enforcement [58] and is considered to have ethological relevance to human declarative memory [53, 59] impaired in schizophrenia. Furthermore, a recent translational study showed that the NOR discrimination ratio obtained in rat studies is a valid analogue of the sensitivity of human recognition memory in a visual-paired comparison task [60]. A 4 h inter-trial interval (ITI) prevents rats’ ability to discriminate the novel from familiar object in our two-trial NOR task [61] that has been thoroughly validated. Acute treatment with dopamine D_3_ receptor antagonists, 5-HT_6_ antagonists and mGluR_2/3_ agonists reverse this impairment, while D_2_ receptor antagonists impair performance using a short ITI [49, 57, 61, 62], making it ideal choice to evaluate the effect of S14481. During the social isolation rearing study, a 2 h ITI was used with which group-housed vehicle-treated rats readily discriminate the novel from the familiar object [57]. In studies investigating the effect of drugs on a delay-induced impairment of NOR, a 4 h ITI was utilised so that normal, adult group-housed vehicle-treated rats were unable to discriminate the novel from the familiar object [54]. Twenty-four hours before the test, rats were habituated to the arena (see “Locomotor Activity” for details) for 1 h. On the test day, rats were briefly reacclimatized to the arena for 3 min before being returned to the home cage for 1 min. During the familiarisation trial, two identical objects (plastic bottles, 8 cm high and 5 cm diameter, covered in white masking tape) were placed in opposite corners of the arena (5 cm from the side and 10 cm from the end wall). The rat was returned to the arena for 3 min, and exploration of each identical objects was recorded with stopwatches. After a 2 or 4 h ITI (in the social isolation and dose-response studies, respectively), the rat was returned to the arena with one familiar and one novel object (bottle wrapped with 4 black stripes 1.2 cm in width). During this second, choice, trial exploration of each object was recorded for 3 min. Exploratory behaviour was defined as sniffing, licking, touching and direct attention to the object with active vibrissae while the nose is within 1 cm of the object. Climbing on or chewing the object was not considered as exploration. Object exploration time during the choice trial was used to determine the discrimination ratio (time at novel–time at familiar/total choice trial object exploration).

### Conditioned Freezing Response

CFR is a well-characterised, species conserved, hippocampal, amygdala and cortical dependent learning and memory task [63] utilised to index long-term associative memory of emotional based preference that is impaired in schizophrenia [64]. CFR is classically considered as part of the exemplary paradigms to investigate acute threat within the negative valence domain under the RDoC framework. However, the memory component of this paradigm, as investigated in this study, is clearly part of the cognitive system domain of the RDoC matrix highly pertinent to emotional memory and deficits thereof are seen in ASD and schizophrenia [65]. CFR was performed in a two-chamber shuttle box with light and dark sides (27 × 25 × 25 cm), separated by an automated door (8 × 8 cm) and a wire grid floor (PanLab, Slab, Barcelona, Spain) using a 3 day test protocol described previously [45, 66]. On the first (conditioning) day, rats received S18841 (2.5 or 10 mg/kg) or vehicle (1 ml/kg s.c.) 30 min before being placed in the light side of the chamber. The door opened after 30 s and latency to cross into the dark side was recorded using a floor sensor which closed the door (ShutAvoid, software v.1.8.2., Panlab S.L, USA). After 30 s in the dark side, a 5 s conditioning stimulus (CS, light and 3 kHz, 89 dB tone) was followed by the unconditioned stimulus (US, 1 s 0.4 mA footshock during the last second of the CS) through the grid floor. A further two CS/US were delivered at 55 s intervals. Time spent freezing (completely immobile except for respiration in a hunched posture with inactive vibrissae) between shocks was recorded by stopwatch. Twenty-four and 48 h post-conditioning, rats were returned to the dark side for 300 s without CS/US and time spent freezing recorded again as an index of associative learning and memory. During the extinction trial (48 h post-conditioning), the CS alone was presented after 300 s and freezing recorded for a further 300 s. Behaviour was also recorded through a camera in the roof of the CFR chamber using Ethovision (Noldus) to enable re-assessment if required.

### Microinjection for NOR

In order to ascertain the importance of glycine receptors in the prefrontal cortex (PFC) in the cognitive effects of S14481, it was delivered by microinjection into this area. The PFC was selected because of the microdialysis data showing changes in either serine or glycine overflow occurred therein with administration of all the glycine agonists, partial agonists and reuptake inhibitors used herein. Lister hooded rats were anaesthetised with isoflurane (3.5% induction and maintained with 2% in 33% O_2_ and 66% N_2_O), placed in a stereotaxic frame to implant cannulae as described before [54]. Two 22-gauge guide cannulae, 1.5 mm apart projecting 3.5 mm, were implanted above the PFC or in separate rats 5.0 mm apart projecting 5.0 mm above the striatum (Plastics One) at the following coordinates from bregma: PFC; AP + 3.0, L ± 0.7, DV − 2.3 and striatum; AP + 0.5, L ± 2.5, DV − 4.0 [50]. Stylets (Plastics One) placed in the cannulae prevented occlusion. Rats were housed individually and allowed to recover for at least 1 week and handled daily to minimise stress associated with drug infusion. On the test day, rats were lightly restrained, stylets removed and sterile 28-gauge injectors (Plastics One) used to bilaterally infuse 1 μl drug or vehicle over 2 min with an infusion pump (Harvard Apparatus, Holliston, MA), 5 min before the NOR familiarisation trial. After behavioural testing, brains were removed and stored in paraformaldehyde for histological verification of the injection from coronal brain sections according to Paxinos and Watson [50].

## Materials

S18841 (3-Hydroxy-4-imidazolidinone), glycine, D-serine, D-cycloserine, sarcosine and ORG24598 were dissolved in 0.9% (*w*/*v*) saline, pH normalized where appropriate and injected in a volume of 1 ml/kg s.c. 30 min prior to each task except for glycine which was dosed at a volume of 4 ml/kg. L701,324 (7-chloro-4-hydroxy-3-(3-phenoxy) phenyl-2(H)-quinolinone, 5 mg/kg in 4 ml/kg) was suspended in distilled water with a few drops of Tween 80. CPP (3-(2-carboxypiperazin-4-yl)propyl-1-phosphonic acid, 10 mg/kg in 1 ml/kg) was dissolved in 0.9% (*w*/*v*) saline and pH normalised with NaOH. L701,324 and CPP were injected i.p. 45 min prior to the NOR task. For microinjection into the PFC or striatum, S18841 was dissolved in artificial cerebrospinal fluid (aCSF) containing 10% *w*/*v* hydroxypropyl-β-cyclodextrin. The rat was lightly restrained and vehicle (1 μl/side) or S18841 injected at a rate of 0.5 μl/min 5 min prior to the behavioural analysis.

### Statistical Analysis

In the social recognition task, the difference “T2–T1” was calculated and dose-response curves for promnesic properties analysed by one-way ANOVA followed by Dunnett’s test. The time course of locomotor activity and rears and time spent exploring each object during the NOR choice trial were analysed using two-way repeated measures RM-ANOVA. Total activity counts and object exploration and discrimination ratio during NOR were analysed with two-way ANOVA (but one-way ANOVA for delay-induced NOR impairment studies). Following ANOVA (*P <* 0.05 was considered significant in all cases), Fishers LSD or Dunnett’s tests were used for post hoc analyses. All statistical analyses were performed using GraphPad Prism v7.0 for Windows (GraphPad Software, San Diego, CA, USA) and SPSS (IBM SPSS Statistics v21, USA), and all data shown are mean ± SEM. For microdialysis, time-course effects of drugs on amino acid levels was analysed by two-way ANOVA, with treatment as a between-subjects factor and time a repeated measure, followed by Dunnett’s post hoc tests. Dose effects of drugs were evaluated by comparing the area under the curve during the first 180 min after i.p. injection of the drug or vehicle and statistical analysis was carried out by a two-way ANOVA followed by Bonferroni’s post hoc tests.

## Results

### Effect of Glycine Modulatory Drugs on PFC Dialysate Levels of Amino Acids and Monoamines in Freely Moving Rats

**Fig. 1:**
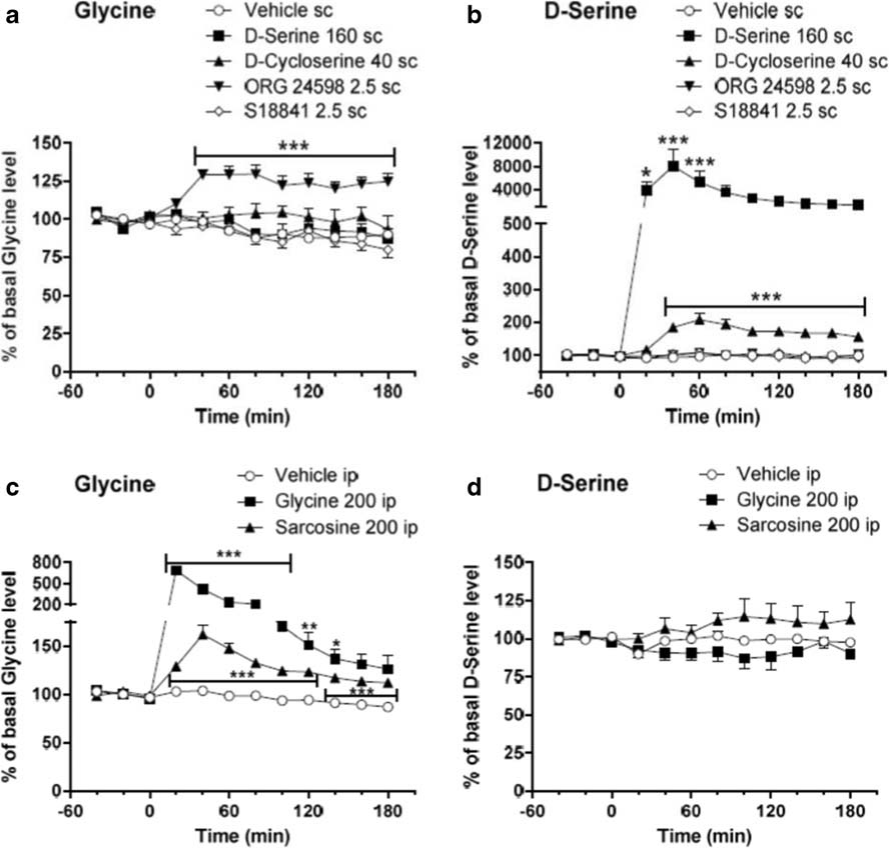
Effect of glycine modulatory site agonists and glycine transport inhibitors on extracellular glycine (**a** and **c**) and D-serine (**b** and **d**) levels in the prefrontal cortex of freely moving male Wistar rats. Microdialysis samples were taken for 180 min at 20-min intervals following acute systemic injection of S18841, D-cycloserine, ORG 24598, D-serine (all s.c.), glycine and sarcosine (both i.p.). Data is expressed as percentage change from a 40-min baseline taken prior to injection (mean ± SEM, *n* = 5–26). Basal levels of glycine and D-serine were 8.28 ± 1.07 and 0.90 ± 0.13 μM, respectively. Two-way ANOVA for **a**, **b** and **c** (not **d**) showed significant main effects of drug (*P* = 0.001), time (*P* = 0.001) and an interaction (*P* = 0.001). Significant two-way ANOVA was found in **a**: (effect on glycine levels) for ORG 24598: main effect of time (*P <* 0.001) and drug (*P <* 0.001) and interaction (*P <* 0.001); in **b**: (effect on D-serine levels) for D-cycloserine: main effect of time (*P <* 0.001), drug (*P <* 0.001) and interaction (*P <* 0.001) and D-serine: main effect of time (*P <* 0.001), drug (*P <* 0.001) and interaction (*P <* 0.001); and in **c**: (effect on glycine) for glycine: main effect of time (*P <* 0.001), drug (*P <* 0.001) and interaction (*P <* 0.001) and sarcosine: main effect of time (*P <* 0.001), drug (*P <* 0.001) and interaction (*P <* 0.001). *P < 0.001, **P < 0.01, ***P < 0.001 between drug and vehicle at that time, Bonferroni’s multiple comparisons post hoc test

Given the pivotal role of PFC function in the modulation of working memory and the cognitive deficits seen in common psychiatric disorders, we used microdialysis to ascertain any common mode of action of the glycine-modifying drugs on neurotransmitter release therein. In freely moving rats, compared to vehicle, both glycine (*F*_(1,19)_ = 306.7, *P <* 0.001) and the two GlyT1 inhibitors (sarcosine *F*_(1,18)_ = 710.3, *P <* 0.0001; and ORG24598, *F*_(1,29)_ = 38.62, *P <* 0.001) significantly increased (by 700, 150 and 130% above basal) glycine in PFC dialysates which peaked at 20 to 40 min (Fig. 1 a and c). In contrast, glycine levels were unaffected by the glycine site agonists, D-serine or D-cycloserine or by S18841. D-serine (*F*_(1,9)_ = 10.00, *P* = 0.0115) and D-cycloserine (*F*_(1,10)_ = 42.99, *P <* 0.001) were the only compounds to significantly elevate PFC D-serine levels (Fig. 1 b and d). No compound changed dialysate levels of glutamate, GABA, 5-HT or acetylcholine and only glycine and sarcosine elevated dopamine while glycine also elevated levels of noradrenaline (Table 1).

**Table 1:** Effect of pharmacological agent on microdialysis amino acid and neurotransmitter levels in the prefrontal cortex of conscious rats. Data (mean + SEM (*n*)) for vehicle and drugs presented as area under the curve expressed as % min/1000; basal values of amino acids are given in μM while those for NE, DA, 5-HT and ACh are in nM. Doses are mg/kg. Significant changes in italic font, *P < 0.05, **P < 0.01, ***P < 0.001 from the vehicle-treated control administered by the same injection route. GlyT1 I, glycine transporter-1 inhibitor; agonist, partial agonist and antagonist refer to the activity at the glycine modulatory site

Drug (dose, route)	Activity	Glycine	D-Serine	L-Serine	Glutamate	GABA	NE	DA	5-HT	ACh
*Basal (μM)*		*8.28 ± 1.07*	*0.90 ± 0.13*	*12.62 ± 0.13*	*1.77 ± 0.26*	*0.06 ± 0.01*	*0.43 ± 0.06*	*0.39 ± 0.03*	*0.23 ± 0.02*	*0.75 ± 0.13*
Vehicle (s.c.)	Control	16.54 ± 0.55 (26)	17.53 ± 0.49 (6)	18.18 ± 0.28 (6)	16.51 ± 0.52 (18)	16.65 ± 0.66 (23)	20.6 ± 1.4 (11)	18.1 ± 0.6 (13)	16.7 ± 0.5 (6)	19.1 ± 0.9 (8)
Vehicle (i.p.)	Control	17.29 ± 0.17 (15)	17.71 ± 0.21 (6)	17.43 ± 0.26 (6)	16.15 ± 0.47 (12)	15.52 ± 0.68 (13)	19.7 ± 0.6 (11)	18.1 ± 0.6 (11)	16.9 ± 0.7 (11)	17.8 ± 1.6 (10)
S18841 (2.5 s.c.)	Partial agonist	16.15 ± 0.48 (8)	17.78 ± 0.31 (4)	17.98 ± 1.48 (4)	17.40 ± 0.83 (8)	13.96 ± 1.06 (8)	18.9 ± 1.1 (6)	18.5 ± 1.1 (6)	15.2 ± 0.3 (6)	20.5 ± 1.1 (7)
D-Cycloserine (40 s.c.)	Partial agonist	18.29 ± 0.60 (6)	30.29 ± 1.23 (6)***	19.33 ± 0.43 (6)	18.00 ± 1.15 (6)	17.24 ± 1.41 (6)	19.4 ± 0.8 (6)	19.1 ± 0.7 (5)	16.7 ± 2.2 (4)	–
D-Serine (160 s.c.)	Agonist	17.06 ± 0.50 (6)	571.12 ± 131.79 (5)***	17.84 ± 0.39 (7)	16.50 ± 0.87 (6)	16.79 ± 1.39 (6)	21.2 ± 1.3 (4)	20.3 ± 0.5 (4)	18.1 ± 2.3 (4)	18.1 ± 3.2 (4)
Glycine (200 i.p.)	Agonist	45.02 ± 2.64 (6)***	16.47 ± 0.52 (5)	20.06 ± 0.30 (9)	14.53 ± 0.85 (6)	17.49 ± 1.68 (6)	25.3 ± 1.7 (8)*	25.7 ± 2.4 (8)*	17.1 ± 1.3 (6)	23.0 ± 1.7 (6)
ORG 24598 (2.5 s.c.)	GlyT1 I	22.03 ± 0.48 (5)***	17.81 ± 0.59 (5)	17.37 ± 0.50 (5)	16.87 ± 0.30 (5)	18.16 ± 1.48 (5)	20.4 ± 1.2 (5)	20.0 ± 1.7 (5)	17.9 ± 2.2 (4)	21.0 ± 1.3 (6)
Sarcosine (200 i.p.)	GlyT1 I	23.07 ± 0.42 (5)**	19.51 ± 0.84 (6)	21.24 ± 0.43 (6)	17.86 ± 0.34 (5)	18.56 ± 1.21 (5)				
Sarcosine (800 i.p.)	GlyT1 I	28.90 ± 2.30 (5)*	18.6 ± 0.5 (5)	24.5 ± 0.9 (5)*	16.0 ± 1.0 (5)	18.6 ± 1.0 (5)	21.8 ± 0.9 (8)	22.3 ± 1.7 (8)*	16.7 ± 0.9 (8)	20.8 ± 2.6 (3)
L701,324 (10 i.p.)	Antagonist	16.14 ± 0.25 (4)	17.87 ± 0.24 (4)	16.80 ± 0.12 (4)	15.29 ± 0.29 (4)	16.17 ± 0.21 (4)	22.5 ± 2.4 (5)	22.7 ± 2.0 (5)	18.8 ± 1.8 (5)	20.0 ± 1.6 (10)

### Effect of Glycine Modulatory Drugs on Disruption of Social Recognition by Scopolamine

**Fig. 2:**
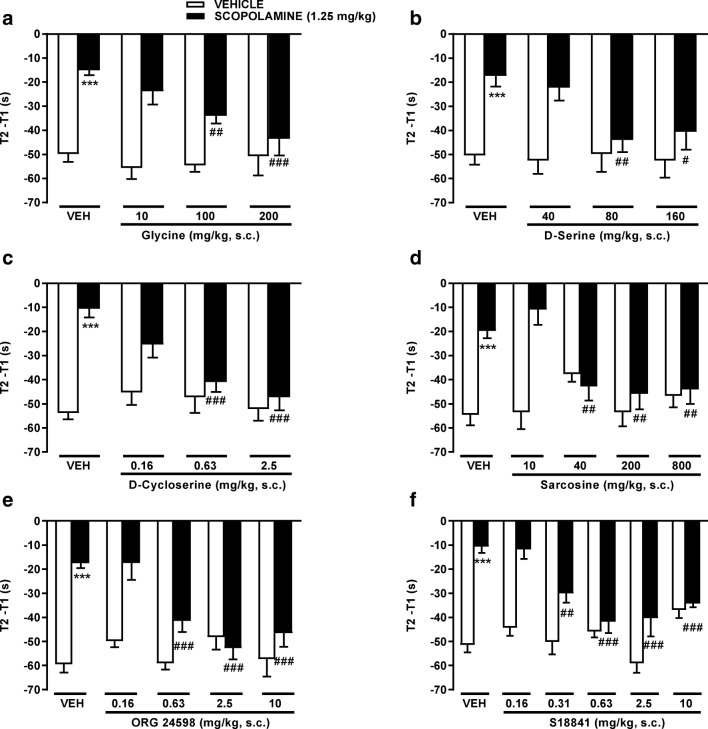
Reversal of a scopolamine-induced deficit in social recognition in Wistar rats by **a** glycine, **b** D-serine, **c** D-cycloserine, **d** sarcosine, **e** ORG 24598 and **f** S18841. Data shown (T2-T1) is the time difference (s) in the exploration by the adult of the juvenile rat between the second (T2) and first (T1) trials. Values are means ± SEM, *n* = 5–13. Rats were sequentially administered test drug (at doses indicated, s.c.) or vehicle (0.9% (*w*/*v*) saline, 1 ml/kg) 30 min prior to T1. Two-way ANOVA showed **a** for glycine: main effect of scopolamine (*P <* 0.001), glycine (*P* = 0.0135) and an interaction (*P* = 0.0170); **b** D-serine: main effect of scopolamine (*P <* 0.0001), D-serine (*P* = 0.0644) and interaction (*P* = 0.045); **c** D-cycloserine: main effect of scopolamine (*P <* 0.0001), D-cycloserine (*P* = 0.0015) and interaction (*P* = 0.0004); **d** sarcosine: main effect of scopolamine (*P <* 0.0001), sarcosine (*P* = 0.0274) and interaction (*P* = 0.0001); **e** ORG 24598: main effect of scopolamine (*P <* 0.0001), ORG 24598 (*P* = 0.0002) and interaction (*P <* 0.0001); and **f** S18841: main effect of scopolamine (*P <* 0.0001), S18841 (*P <* 0.0001) and interaction (*P <* 0.0001). ***P < 0.001 between vehicle/scopolamine and vehicle/vehicle; ^#^*P <* 0.05, ^##^*P <* 0.01 and ^###^*P <* 0.001 between drug/scopolamine and vehicle/scopolamine Newman-Keuls post hoc test

We next compared the ability of all the glycine-modifying drugs to reverse a cholinergic-induced impairment of social discrimination of a novel from a familiar juvenile rat, a cortical-dependent working memory task to examine the functional consequence of the microdialysis data in adult rats. Social recognition was selected because of its perceived translational relevance to dysfunction in psychiatric disorders [53]. In the social recognition task with no intertrial delay, vehicle rats investigated the juvenile rat significantly less in T2 than T1, demonstrating social recognition memory (Fig. 2). However, adults given scopolamine (1.25 mg/kg s.c.) prior to T1 spent a similar time investigating the juvenile in T2 and T1, indicative of impaired recognition memory compared to vehicle controls. The glycine site agonists, glycine (Fig. 2a, main effect of scopolamine, *F*_(1,50)_ = 53.14, *P <* 0.001; glycine, *F*_(3,50)_ = 3.93, *P* = 0.0135 and an interaction, *F*_(3,50)_ = 3.73, *P* = 0.0170), D-serine (Fig. 2b, scopolamine, *F*_(1,52)_ = 24.32, *P <* 0.0001; D-serine, *F*_(3,52)_ = 2.57, *P* = 0.0644 and interaction, *F*_(3,52)_ = 2.66, *P* = 0.045), D-cycloserine (Fig. 2c, scopolamine, *F*_(1,51)_ = 31.59, *P <* 0.0001; D-cycloserine, *F*_(3,51)_ = 5.93, *P* = 0.0015 and interaction, *F*_(3,51)_ = 7.22, *P* = 0.0004), and S18841 (Fig. 2f, scopolamine, F_(1,581)_ = 69.92, *P <* 0.0001; S18841, *F*_(5,81)_ = 7.76, *P <* 0.0001 and interaction, *F*_(5,81)_ = 6.85, *P <* 0.0001) and the GlyT1 inhibitors, sarcosine (Fig. 2d, scopolamine, *F*_(1,61)_ = 21.55, *P <* 0.0001; sarcosine, *F*_(4,61)_ = 2.94, *P* = 0.0274 and interaction, *F*_(4,61)_ = 6.90, *P* = 0.0001) and ORG24598 (Fig. 2e, scopolamine, *F*_(1,62)_ = 45.08, *P <* 0.0001; ORG24598, *F*_(4,62)_ = 6.48, *P* = 0.0002 and interaction, *F*_(4,62)_ = 7.76, *P <* 0.0001) all reversed the impairment in social recognition caused by scopolamine, restoring the reduction in time spent exploring the juvenile in T2. Of particular note for every compound, the lowest dose to significantly increase social discrimination was lower than or equal to that shown to elevate glycine or D-serine levels in PFC microdialysates (Table 2). Furthermore, reversal of the recognition impairment occurred irrespective of whether the drug elevated glycine or D-serine, suggesting that either mechanism was effective, as is partial agonism at the glycine site.

**Table 2:** Comparison of the dose of each glycine modulatory site agonist, partial agonist or GlyT1 inhibitor (GlyT1 I, as indicated in the activity column) to significantly (a) elevate prefrontal cortex (PFC) glycine and D-serine, (b) reverse scopolamine-induced impairment in social recognition (taken from T_2_-T_1_ active social investigation time, s) and (c) prevent the 4 h intertrial time delay-induced reduction in NOR (taken from choice trial discrimination ratio, (novel-familiar/total object exploration)). **P < 0.01 and ***P > 0.001 from vehicle control in that group of rats Dunnett’s post hoc test following ANOVA. NOR = novel object recognition, i.p. = intraperitoneal and s.c. = subcutaneous

Drug	Activity	Elevation in PFC microdialysate glycine or D-serine dose mg/kg and route of injection		Reversal of scopolamine-impaired social recognition dose mg/kg s.c.	Reversal of time-delay reduction in NOR dose mg/kg i.p.
		Glycine	D-serine		
Glycine	Agonist	200 i.p.***	–	100**	160*
D-Serine	Agonist	–	160 s.c. ***	80**	40**
D-Cycloserine	Partial agonist	–	40 s.c. ***	0.63***	2.5*
Sarcosine	GlyT1 I	200 i.p.***	–	40**	200*
ORG24598	GlyT1 I	2.5 s.c.***	–	0.63***	2.5**
S18841	Partial agonist	–	–	0.31 **	10**

### Effect of Glycine Modulatory Drugs on the Delay-Induced Impairment of Novel Object Recognition

**Fig. 3:**
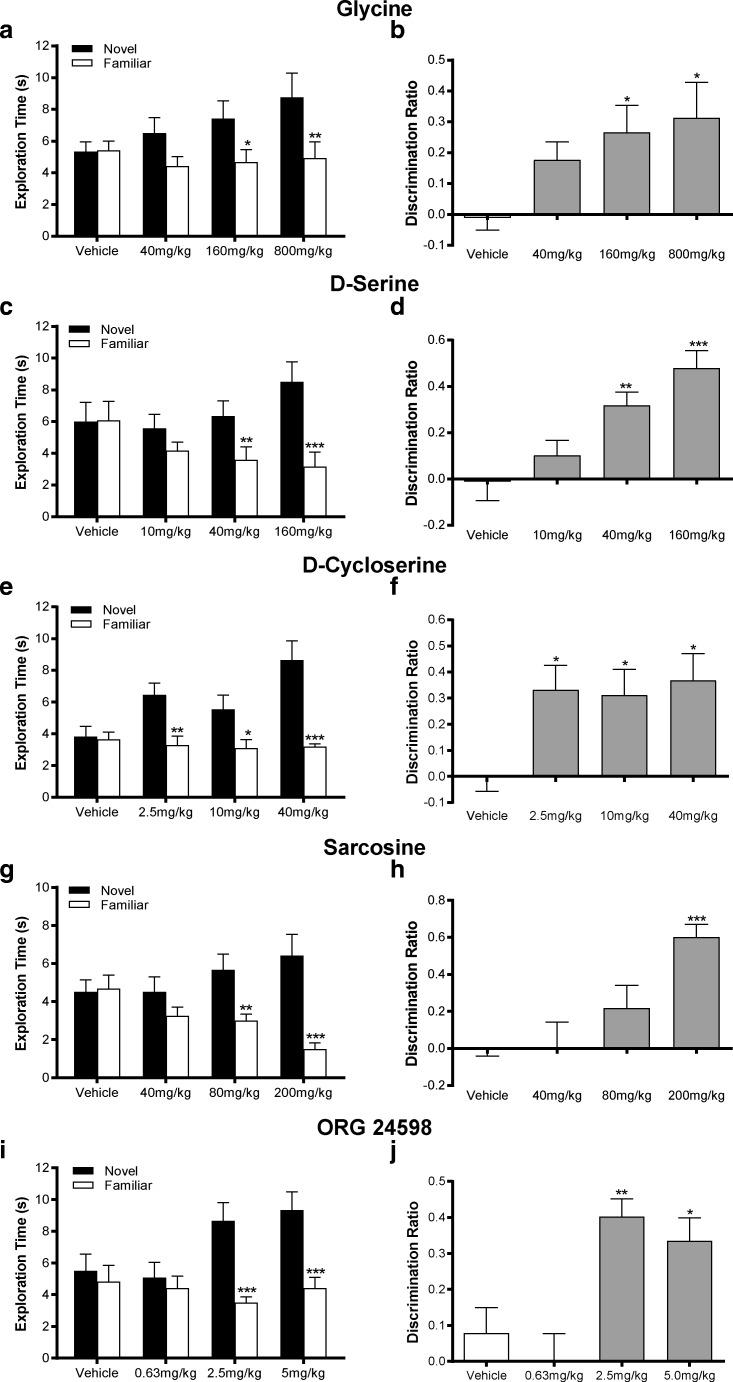
Ability of glycine receptor agonists (**a**–**f**) and glycine reuptake inhibitors (**g**–**j**) to reverse natural forgetting in a 4-h intertrial interval novel object recognition paradigm. Comparison of the effect of glycine (**a** and **b**, 40–800 mg/kg, 4 ml/kg), D-serine (**c** and **d**, 10–40 mg/kg, 1 ml/kg), D-cycloserine (**e** and **f**, 2.5–40 mg/kg, 1 ml/kg), sarcosine (**g** and **h**, 40–200 mg/kg, 1 ml/kg) and ORG 24598 (**i** and **j**, 0.63–5 mg/kg, 1 ml/kg) injected s.c. 30 min prior to the familiarisation trial in Lister hooded rats (*n* = 12 unless stated, data shown as mean ± SEM). **a**, **c**, **e**, **g**, **i** Actual novel and familiar object exploration times (s) during the second choice trial; *P < 0.05, **P < 0.01, ***P < 0.001 significantly different from novel object in same treatment group, Bonferroni post hoc following repeated measures ANOVA. **b**, **d**, **f**, **h**, **j** Derived discrimination ratio (novel object exploration–familiar object exploration/total object exploration); *P < 0.05, **P < 0.01, ***P < 0.001 significantly different from vehicle control, Dunnett’s post hoc. In **a**, RM-ANOVA showed that the object × glycine treatment interaction just failed to reach significance (*P* = 0.057). **b** ANOVA showed a significant main effect of glycine treatment (*P <* 0.05). **c** Significant object × D-serine interaction (*P <* 0.001). **d** Significant main effect of D-serine (*P <* 0.001). **e** Significant object × D-cycloserine interaction (*P <* 0.01, *n* = 11). **f** Significant effect of D-cycloserine (*P <* 0.05, *n* = 11). **g** Significant object × sarcosine interaction (*P <* 0.001). **h** Significant main effect of D-serine treatment (*P <* 0.001). **i** Significant object × ORG 24598 treatment interaction (*P <* 0.001). **j** Significant effect of ORG 24598 treatment (*P <* 0.001)

NOR is a visual learning and memory task considered to have ethological relevance to human declarative memory [53, 59] that is both PFC-dependent and impaired in schizophrenia, and its selection is thoroughly considered in the discussion. We therefore performed dose-response studies with all the glycine-modifying compounds to establish their ability to improve a time-delay-induced impairment in NOR. With vehicle injection in all studies, rats were unable to distinguish the novel from the familiar object in the choice trial (Fig. 3 a, c, e, g and i). However, the highest two doses of every agonist or glycine reuptake inhibitor increased the time spent exploring the novel over the familiar object, and even the lowest dose (2.5 mg/kg) of D-cycloserine produced a significant increase. With glycine, D-serine and sarcosine (*F*_(3, 44)_ = 2.709, *P* = 0.057, *F*_(3, 44)_ = 7.45, *P <* 0.001, and *F*_(3, 44)_ = 10.27, *P <* 0.001, respectively), the significant increase in novel object exploration was dose-related, while the improvement seen with D-cycloserine (*F*_(3, 44)_ = 6.518, *P <* 0.01) was similar at all three doses and with ORG24598 (*F*_(3, 44)_ = 8.23, *P* < 0.001) only apparent at the highest two doses.

Analysis of the choice trial discrimination ratio data (Fig. 3 b, d, f, h and j) from the same rats confirmed improvement in object discrimination predicted from raw exploration times. In all groups, the discriminated ratio was at or close to chance following vehicle and significantly increased with glycine (*F*_(3, 44)_ = 3.17, *P <* 0.05; *P <* 0.05 two highest doses), D-serine (*F*_(3, 44)_ = 9.64, *P <* 0.001; *P <* 0.01 and *P <* 0.001 with 40 and 160 mg/kg, respectively), D-cycloserine (*F*_(3, 44)_ = 3.73, *P <* 0.05; *P <* 0.05 with all doses), sarcosine (*F*_(3, 44)_ = 7.88, *P <* 0.001; *P <* 0.001, highest dose only) and ORG24598 (*F*_(3, 44)_ = 8.72, *P <* 0.001; *P <* 0.01 and *P <* 0.05 at 2.5 and 5 mg/kg, respectively). Thus, as with social recognition, the minimum dose to improve NOR was equivalent to, or lower than, that found to elevate either PFC glycine or D-serine in microdialysates (Table 2), consistent with mechanistic association.

No dose of any drug caused a preferential exploration of either object during the familiarisation trial (Table 3), although with sarcosine ANOVA showed a significant (*F*_(3, 44)_ = 10.27, *P <* 0.001) location × drug interaction. Furthermore, only in the D-serine group was there any main effect of drug on total object exploration time in the familiarisation trial (*F*_(3, 44)_ = 2.98, *P <* 0.05) and this was not dose-related. In all other groups, there was no effect of drug treatment on total exploration in either familiarisation or choice trials (Table 3). Therefore, alteration of choice trial object exploration by all five drugs resulted from redistribution of attention towards the novel object, consistent with a specific effect on visual learning and memory and not a reduction in attention to both objects.

**Table 3:** Object exploration during familiarisation and choice trials with glycine agonists and GlyT1 inhibitors, accompanying Fig. 3. Time (s, mean (SEM)) spent exploring (from left to right) front and back object and both objects during the (first) familiarisation trial and total time exploring both objects in the (second) choice trial in the novel object recognition test. For each glycine site agonist or glycine transporter-1 inhibitor, three doses (as stated) were compared to the effect of vehicle (s.c.) given in a balanced quasi-random order over 4 weeks in five separate groups of group-housed male Lister Hooded rats. Following D-serine administration there was a significant main effect of drug treatment on total object exploration during the familiarisation trial (*P* < 0.05). There was no preference for the location of the object during the familiarisation trial in any drug group, but following treatment with sarcosine there was a significant location x drug interaction (*P* < 0.05). There was no significant effect of any drug on total exploration during the choice trial (all *P* > 0.05). *P < 0.05 from vehicle at that time point, Dunnett’s post hoc following ANOVA

		Familiarisation trial		Choice trial	
Drug	Dose	Front object (s)	Back object (s)	Total exploration (s)	Total exploration (s)
Glycine (*n* = 12)	Vehicle 40 mg/kg 160 mg/kg 800 mg/kg	5.5 (0.4) 6.9 (0.9) 7.0 (0.8) 6.9 (0.9)	5.7 (0.6) 8.0 (1.0) 6.2 (0.6) 6.3 (0.9)	11.2 (0.8) 14.9 (1.8) 13.2 (1.2) 13.2 (1.6)	10.8 (1.1) 10.9 (1.5) 12.1 (1.6) 13.7 (2.1)
D-Serine (*n* = 12)	Vehicle 10 mg/kg 40 mg/kg 160 mg/kg	5.8 (1.0) 6.7 (0.7) 9.0 (0.9) 5.7 (0.9)	5.5 (0.8) 7.9 (0.8) 8.6 (1.3) 6.7 (0.6)	11.3 (1.7) 14.6 (1.2) 17.7 (2.2)* 12.3 (1.3)	12.1 (2.3) 9.8 (1.3) 9.9 (1.7) 11.7 (1.9)
D-Cycloserine (*n* = 11)	Vehicle 2.5 mg/kg 10 mg/kg 40 mg/kg	7.5 (0.9) 6.3 (0.6) 5.5 (0.7) 6.5 (0.8)	7.6 (0.8) 6.5 (0.6) 6.7 (0.7) 6.9 (1.4)	15.1 (1.5) 12.7 (1.0) 12.2 (1.2) 13.5 (2.2)	7.5 (1.1) 9.7 (1.1) 8.6 (1.3) 11.8 (1.2)
Sarcosine (*n* = 12)	Vehicle 40 mg/kg 80 mg/kg 200 mg/kg	6.0 (0.7) 4.5 (0.4) 5.1 (0.6) 5.5 (0.4)	4.1 (0.4) 4.8 (0.6) 5.3 (0.4) 5.6 (0.5)	10.1 (1.0) 9.3 (0.8) 10.3 (0.8) 11.1 (0.7)	9.2 (1.3) 7.8 (1.2) 8.7 (1.1) 7.9 (1.3)
ORG 24598 (*n* = 12)	Vehicle 0.63 mg/kg 2.5 mg/kg 5 mg/kg	5.0 (0.8) 5.9 (1.0) 8.4 (1.2) 6.9 (0.9)	6.1 (0.8) 7.2 (1.0) 7.4 (1.1) 6.9 (0.7)	11.1 (1.5) 13.1 (1.6) 15.8 (2.1) 13.8 (1.3)	10.3 (2.0) 9.5 (1.7) 12.2 (1.4) 13.8 (1.5)

**Fig. 4:**
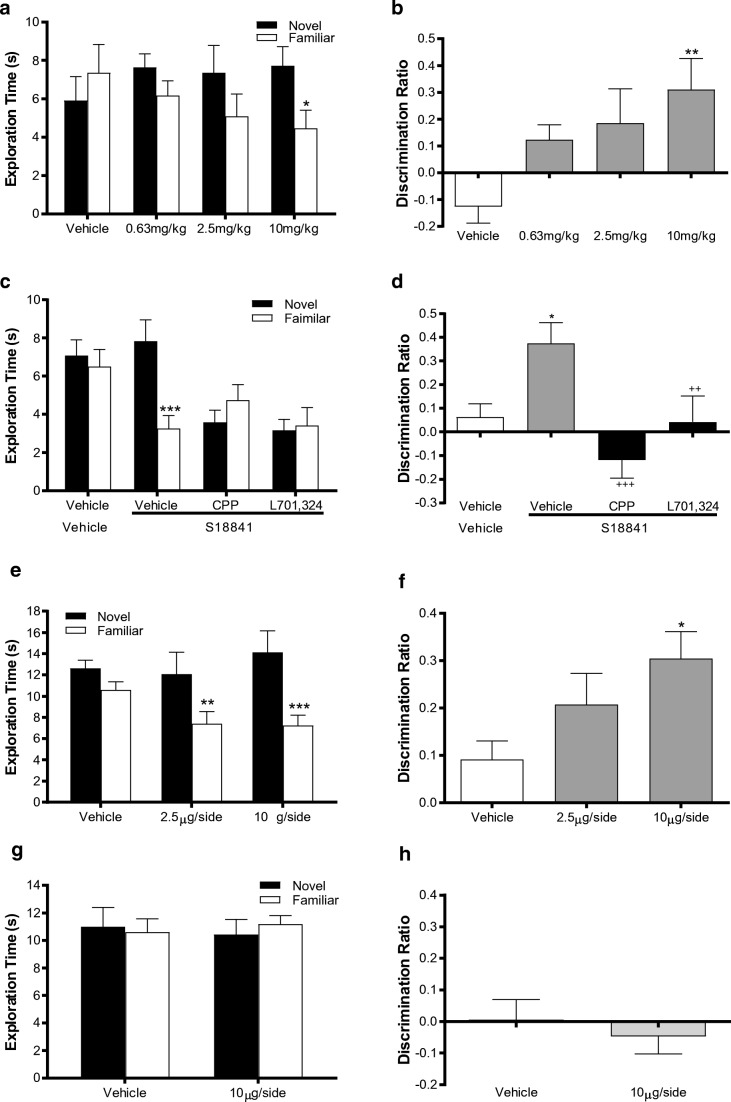
Restoration of a time-delay impairment (natural forgetting) in novel object recognition by the partial glycine agonist, S18841, is prevented by the glycine antagonist, L701,324, and the NMDA receptor antagonist, CPP. S18841 (in **a** and **b** 0.63–10 mg/kg, in **c** and **d** 10 mg/kg) or vehicle (1 ml/kg) injected s.c. 30 min prior to the familiarisation trial in Lister hooded rats. CPP (20 mg/kg, 1 ml/kg), L701,324 (10 mg/kg, 4 ml/kg) or vehicle (1 ml/kg) injected i.p. 20 min prior to the familiarisation trial in **c** and **d**. The partial glycine agonist, S18841, delayed natural forgetting in the novel object discrimination paradigm when discretely injected into the prefrontal cortex (**e** and **f**) but not the striatum (**g** and **h**). S18841 (2.5 μg or 10 μg/side) or vehicle (1 μl/side) injected 5 min prior to the familiarisation trial in group-housed male Lister hooded rats. Data presented as mean ± SEM. **a**, **c**, **e**, **g** Actual novel and familiar object exploration time (s) during the second choice trial. **b**, **d**, **f**, **h** Derived discrimination ratio (novel object exploration–familiar object exploration/total object exploration). In **a**, RM-ANOVA showed a significant object × S18841 treatment interaction (*P <* 0.05), *P < 0.05 from novel object in same treatment group, Bonferroni post hoc. **b** ANOVA showed a significant effect of S18841 treatment (*P <* 0.05), **P < 0.01 from vehicle, Dunnett’s post hoc. **c** RM-ANOVA significant object × S18841 treatment (*P <* 0.01) and object × CPP/L701,324 interactions (*P <* 0.001), ***P < 0.001 from novel object in same treatment group, Bonferroni post hoc*.***d** ANOVA showed a significant effect of S18841 treatment (*P <* 0.05) and a significant effect of CPP/L701,324 (*P <* 0.01), **P < 0.01 from vehicle**/**vehicle; ^++^*P <* 0.01, ^+++^*P <* 0.001 from S18841/vehicle, Fishers LSD post hoc. **e** RM-ANOVA showed a significant object × S18841 treatment interaction (*P <* 0.05), **P < 0.01, ***P < 0.001 from novel object in same treatment group, Bonferroni post hoc. **f** ANOVA showed a significant effect of S18841 treatment (*P <* 0.05), *P < 0.05 from vehicle, Dunnett’s post hoc. **g** RM-ANOVA no significant object × S18841 treatment interaction (*P* = 0.510). **h**, ANOVA showed a no significant effect of S18841 treatment (*P* = 0.531)

Acute pretreatment with the glycine site partial agonist, S18841 (0.63 to 10 mg/kg s.c.), reversed a time delay-induced impairment in NOR (Fig. 4 a and b). With a 4 h ITI, vehicle-treated rats were unable to distinguish the novel from the familiar object in the choice trial (Fig. 4a) as expected [54]. S18841 produced a dose-related restoration of preferential novel object exploration (Fig. 4a object × S18841; *F*_(3, 40)_ = 3.02, *P <* 0.05), confirmed by the discrimination ratio (Fig. 4b, *F*_(3, 40)_ = 3.70, *P <* 0.05) being significantly (*P <* 0.01) greater than vehicle with the highest dose (10 mg/kg). There was no preference for either object and no main effect of drug treatment during the familiarisation trial (Table 4). Similarly S18841 had no effect on total object exploration during either the familiarisation (Table 4) or choice trials confirming a specific pro-cognitive effect of S18841.

**Table 4:** Object exploration during familiarisation and choice novel object recognition trials accompanying Figs. 3 and 4. As detailed in Table 2 legend, object exploration times (s) in the familiarisation and choice trials during the novel object recognition task shown as mean (SEM). In descending order, the groups show (1) the dose-response to systemic administration of the glycine site partial agonist, S18841 alone, (2) S18841 combined with administration of either the NMDA receptor antagonist CPP or the glycine site antagonist L701,324, or S18841 given by discrete microinjection into either the (3) medial prefrontal cortex (PFC) or (4) the striatum. None of the drugs had any effect on either the total object exploration or distribution of object exploration during the familiarisation trial in any of the studies (all *P* > 0.05). Treatment with CPP/ L701,324 significantly reduced total familiarisation trial exploration (*P* < 0.05), and in that study, there was also an effect of S18841 treatment (*P* < 0.05). *P < 0.05, **P < 0.01, ***P < 0.001 significantly different from vehicle/vehicle group at that time point; ^+^*P* < 0.05 significantly different from S18841/vehicle group at that time point, Fisher LSD post hoc. PFC, prefrontal cortex

Dose of S18841		Familiarisation trial			Choice trial
		Front object (s)	Back object (s)	Total exploration (s)	Total exploration (s)
Vehicle 0.63 mg/kg 2.5 mg/kg 10 mg/kg	(*n* = 11) (*n* = 11) (*n* = 11) (*n* = 11)	8.9 (1.4) 7.8 (1.6) 6.6 (1.4) 5.8 (0.9)	8.5 (1.1) 7.8 (1.3) 7.0 (1.7) 6.2 (0.8)	17.4 (2.4) 15.6 (2.6) 13.6 (2.9) 12.0 (1.6)	13.3 (2.7) 13.8 (1.3) 12.5 (2.1) 12.2 (1.3)
Vehicle/Vehicle S18841^a^/Vehicle S18841^a^/CPP^b^ S18841^a^/L701324^a^	(*n* = 12) (*n* = 12) (*n* = 12) (*n* = 12)	9.4 (0.8) 7.8 (0.9) 5.8 (0.9) 4.5 (0.9)	9.5 (1.0) 6.8 (0.7) 4.8 (0.6) 5.4 (0.7)	18.9 (1.6) 14.7 (1.5)* 10.7 (1.2)** 9.9 (1.3)*** ^+^	13.6 (1.6) 10.5 (1.4) 8.3 (1.4) 6.6 (1.3)
Vehicle, PFC 2.5 μg/side 10 μg/side	(*n* = 16) (*n* = 8) (*n* = 8)	13.5 (1.6) 13.3 (1.2) 12.5 (1.7)	13.1 (1.9) 13.9 (2.2) 12.9 (1.7)	26.5 (3.0) 27.2 (3.0) 25.3 (3.2)	23.2 (1.2) 19.5 (3.0) 21.4 (2.8)
Vehicle, striatum 10 μg/side	(*n* = 8) (*n* = 8)	18.2 (3.2) 17.8 (1.4)	20.4 (3.7) 17.8 (1.8)	38.5 (6.6) 35.6 (2.9)	21.6 (2.1) 21.6 (1.3)

### Prevention of the Pro-cognitive Effect of S18841 on NOR by Blockade of NMDA Receptors

To establish the downstream role of NMDA receptor activation in the effect of S18841 on recognition memory, we showed that the glycine modulatory site antagonist, L701,324, and the NMDA receptor antagonist, CPP, completely prevented the reversal of impairment of NOR by S18841 (Fig. 4 c and d). Akin to data in the dose-response study, vehicle-treated rats were unable to discriminate novel and familiar objects but S18841 (10 mg/kg) restored novel object preference (Fig. 4c object × S18841; *F*_(1, 40)_ = 12.29, *P <* 0.01). Of note, co-treatment with either CPP or L701,324 prevented the S18841-induced reversal (object × CPP/L701,324; *F*_(2, 44)_ = 14.660, *P <* 0.001. Furthermore, the significant increase in discrimination ratio in the S18841/vehicle group (Fig. 4d, *F*_(1, 44)_ = 6.79, *P <* 0.05) was abolished by S18841/CPP and S18841/L701,324 combinations (*F*_(2, 40)_ = 8.82, *P <* 0.01), the ratio being significantly less (*P <* 0.001 and *P <* 0.01, respectively) with CPP and L701,324 than with vehicle/S18841 treatment. Both CPP and L701,324 reduced total object exploration in the familiarisation trial (Table 3; S18841; *F*_(1, 44)_ = 4.56, *P* < 0.05, CPP/L701,324; *F*_(1, 44)_ = 3.29, *P* < 0.05), but there were no significant changes in total choice trial exploration. Despite total exploration being reduced, there was no object preference (Table 4) and neither treatment effected the distribution of object exploration during the familiarisation trial.

### Reversal of Delay-Dependent Impairment of NOR by Intra-PFC S18841

Consistent with the involvement of the PFC in the pro-cognitive effects of S18841, bilateral PFC microinjection of S18841 reversed the delay-dependent impairment of NOR (Fig. 4e, object × S18841; *F*_(2, 29)_ = 4.11, *P <* 0.05) such that rats given either dose spent significantly more (*P <* 0.01 and *P <* 0.001) time exploring the novel object. PFC injection of S18841 also increased the derived discrimination ratio (Fig. 4f, *F*_(2, 29)_ = 4.68, *P <* 0.05) such that it was significantly greater than vehicle (*P <* 0.05). In contrast, injecting S18841 into the striatum at the higher dose failed to enhance exploration of the novel object (Fig. 4g) or alter the discrimination ratio (Fig. 4h). Bilateral microinjections of S18841 into the PFC or striatum had no effect on total object exploration during either the familiarisation or choice trials (Table 4), emphasising that improvement in visual recognition memory was not due to changes in locomotor activity or inattention to the objects during acquisition. In addition, irrespective of the site of S18841 microinjection, it did not alter the distribution of object exploration during the familiarisation trial (Table 4). Histological analysis confirmed the site of the microinjections in all rats (see online resource; Fig. S1).

### Effect of S18841 upon Hyperactivity of Isolation-Reared Rats

**Fig. 5:**
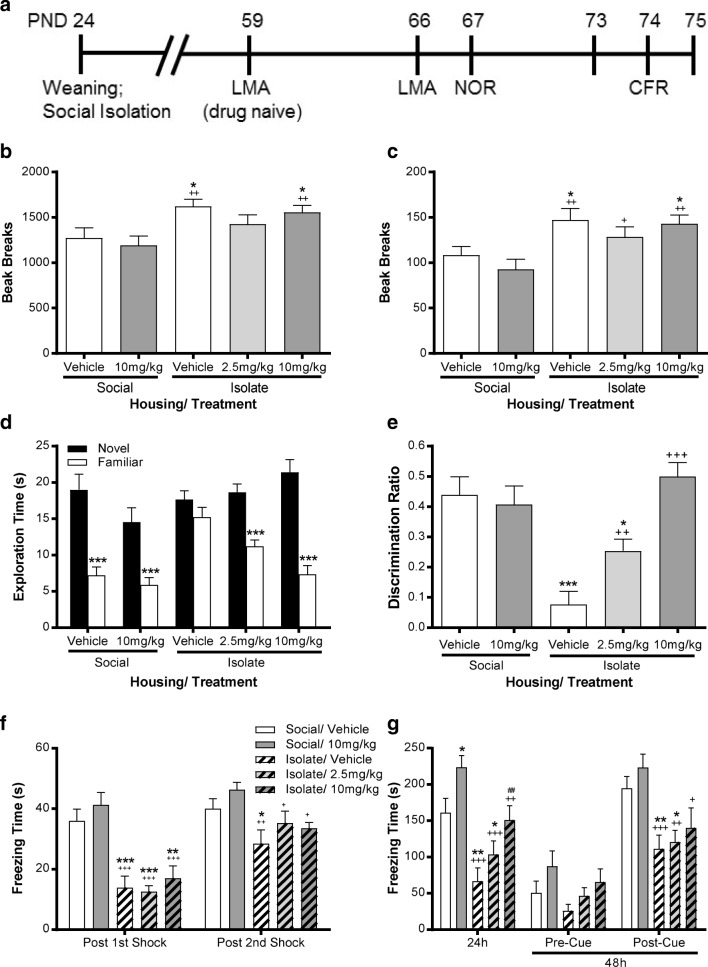
**a** Time course protocol for sequential assessment of locomotor activity (LMA), novel object recognition (NOR) and conditioned freezing in rats placed in individual cages (social isolation) or group-housed (littermate controls) from weaning on post natal day 24. Effect of the partial glycine agonist, S18841, on **b** locomotor activity and **c** number of rears observed over 1 h in a novel arena, **d** novel and familiar object exploration time (s) and **e** the derived discrimination ratio (novel object exploration–familiar object exploration/total object exploration) during the second choice trial of novel object recognition. Also in the conditioned freezing task, the total freezing time (s) **f** after the first two of three foot shocks during conditioning and **g** upon reintroduction into the chamber 24 h after conditioning and again 48 h after conditioning both before (Pre-Cue) and after presentation of the cue alone (Post-Cue), as indicated. Rats were either group-housed (Social) or single housed (Isolate) from weaning on post-natal day 24. S18841 (2.5 or 10 mg/kg s.c.) or vehicle (1 ml/kg) was injected 30 min prior to **b**, **c** being placed in the arena for 1 h, **d**, **e** before the familiarisation trial and **f**, **g** before receiving foot shock conditioning in the associative learning paradigm. Data expressed as mean ± SEM, *n* = 10 per group. In **b**, RM-ANOVA showed a significant main effect of housing (*P <* 0.001) but not drug treatment (*P* = 0.283). *P < 0.05 from social/vehicle rats; ^++^*P <* 0.01 from social/10 mg/kg S18841, Fishers LSD. **c** RM-ANOVA revealed a significant main effect of housing (*P <* 0.001) but not drug treatment (*P* = 0.317). *P < 0.05 from social/vehicle rats; ^+^*P <* 0.05, ^++^*P <* 0.01 from social/10 mg/kg S18841, Fishers LSD. **d** RM-ANOVA showed a significant object × S18841 treatment (*P <* 0.05) and object × S18841 treatment × housing (*P <* 0.001) interaction, ***P < 0.001 from novel object in same treatment group, Bonferroni post hoc. **e** ANOVA revealed a significant main effect of housing (*P <* 0.05) and S18841 treatment (*P <* 0.001) and a significant housing × S18841 interaction (*P <* 0.001), *P < 0.05, ***P < 0.001 from social/vehicle. **f** RM-ANOVA indicated a significant conditioning × housing interaction (*P <* 0.05) and a main effect of housing (*P <* 0.001), *P < 0.05, ***P < 0.001 significantly different from social/vehicle at that time point; ^+^*P <* 0.05, ^++^*P <* 0.01, ^+++^*P <* 0.001 from social/10 mg/kg S18841, Fisher LSD post hoc. **g** At 24 h post-conditioning ANOVA indicated a significant main effect of housing (*P <* 0.001) and S18841 treatment (*P <* 0.05). At 48 h, there was no main effect of housing or S18841 treatment pre-cue but there was a main effect of housing post cue (*P <* 0.001), *P < 0.05, **P < 0.01 from social/vehicle; ^+^*P <* 0.05, ^++^*P <* 0.01, ^+++^*P <* 0.001 from social/10 mg/kg S18841, ^##^*P <* 0.01 from isolate/vehicle Fisher LSD post hoc

Commencing 5 weeks after weaning, rats underwent a battery of behavioural tasks (Fig. 5a), the first being placed in an unfamiliar chamber without drug administration to confirm that isolation induced the expected novelty-induced hyperactivity (supplementary Fig. S2A, *F*_(1, 48)_ = 16.70, *P <* 0.001). Isolation-reared rats showed greater horizontal activity over time (time × housing; *F*_(11, 528)_ = 3.29, *P <* 0.001) when introduced to the chamber compared to their group-housed littermates, confirming development of the ‘isolation syndrome’. In addition to hyperactivity, as expected, isolated rats displayed significantly more rears over 1 h than group-housed controls (online resource; Fig. S2B).

Six weeks after weaning, rats were pretreated with S18841 (2.5 or 10 mg/kg) or vehicle before being re-introduced into the chamber (Fig. 5a). As expected, the socially isolated rats were hyperactive (Fig. 5b, *F*_(1, 45)_ = 14.22, *P <* 0.001) but S18841 had no significant effect on locomotor activity in group-housed or isolated rats. Similarly, the increase in total rears in the isolated rats (Fig. 5c, *F*_(1, 45)_ = 16.42, *P <* 0.001) was unaltered by S18841. This suggests that the partial glycine agonist may have little effect on the augmentation of ventral tegmental dopaminergic activation of the nucleus accumbens thought to contribute to the isolation-induced hyperactive response to a novel environment.

### Reversal of NOR Deficits in Isolation-Reared Rats by S18841

The effect of S18841 on NOR in isolation-reared rats was examined to ascertain if it could reverse a neurodevelopmental impairment in visual recognition memory (Fig. 5 d and e). S18841 reversed the impairment of NOR seen in isolation-reared rats, ANOVA showing an object × S18841 treatment (*F*_(2, 45)_ = 3.84, *P <* 0.05) and object × housing × S18841 treatment (*F*_(1, 45)_ = 22.89, *P <* 0.001) interaction (Fig. 5d). Analysis of the derived discrimination ratio (Fig. 5e) showed a significant main effect of housing (*F*_(1, 45)_ = 7.06, *P <* 0.05), confirming that this index of NOR was also significantly greater in isolated rats treated with either dose of S18841 (2.5 mg/kg; *P <* 0.01, 10 mg/kg; *P <* 0.001) than in vehicle-treated isolates, consistent with the drug-induced reversal of the neurodevelopmental cognitive impairment. Rats did not show any preferential exploration of the identical objects in the familiarisation trial (Table 5), irrespective of housing condition during development or drug treatment. Furthermore, during both familiarisation and choice NOR trials, total object exploration was greater in the isolated groups but unaffected by drug treatment (Table 5). Thus, the improvement in NOR produced by S18841 in isolation-reared rats was not due to increased object attention during acquisition but a preferential improvement in visual recognition memory.

**Table 5:** Object exploration during the familiarisation and choice trials in the novel object recognition task in rat littermates reared from weaning either as a group or in social isolation accompanying Fig. 5 in the manuscript. As detailed in Table 2 legend, object exploration times (s) in the familiarisation and choice trials (separated by a 2 h interval) during the novel object discrimination task shown as mean (SEM), *n* = 10 per group. Rats were injected with either S18841 (2.5 or 10 mg/kg) or vehicle (1 ml/kg) s.c. 30 min prior to start of task. During the familiarisation trial, although there was no significant difference between the exploration of the front and back object, there was a significant location × housing (*P* < 0.01) and location × S18841 treatment (*P* < 0.05) interaction. During both the familiarisation and choice trials, ANOVA showed a main effect of housing (*P* < 0.05 and *P* < 0.01, respectively). However, post hoc analysis revealed that the only significant differences were between the group-housed (social) rats administered S18841 and the isolation-reared rats, ^+^*P* < 0.05, ^++^*P* < 0.01 significantly different from social/10 mg/kg S18841 group during that trial, Fisher LSD post hoc

Housing	Drug	Familiarisation trial			Choice trial
		Front object (s)	Back object (s)	Total exploration (s)	Total exploration (s)
Social Social Isolate Isolate Isolate	Vehicle 10 mg/kg Vehicle 2.5 mg/kg 10 mg/kg	15.9 (1.5) 12.3 (2.0) 13.7 (1.1) 15.4 (1.6) 14.1 (1.9)	14.8 (2.1) 11.3 (2.1) 19.5 (1.4) 14.8 (1.5) 18.8 (1.8)	31.2 (3.5) 25.8 (3.0) 33.2 (1.4)^+^ 30.2 (2.6) 32.9 (3.5)^+^	28.7 (1.7) 22.4 (2.3) 32.9 (2.2)^++^ 29.8 (1.7)^++^ 28.7 (2.4)^+^

### Partial Reversal of CFR Deficits in Isolation-Reared Rats by S18841

As reported previously, social isolation from weaning induces deficits in contextual and conditioned associative learning in the CFR task (Fig. 5 f and g) which is thought to have translational relevance to negative valence systems in man. Neither isolation housing nor S18841 treatment altered the latency of rats to cross from the more aversive light to the dark side (data not shown) during conditioning, indicating that alterations in CFR were not confounded by changes in anxiety or locomotor activity. However, freezing after each shock during conditioning was significantly lower in isolates than group-housed rats (Fig. 5f, housing; *F*_(1, 45)_ = 1.57, *P <* 0.001) but increased with each conditioning shock, as in group-housed controls, and S18841 had no effect. Vehicle-treated isolated rats froze for significantly less time than vehicle-treated group-housed littermates (*P <* 0.01) when placed back in the conditioning context 24 h later (Fig. 5g). ANOVA showed a main effect of housing (*F*_(1, 45)_ = 20.09, *P <* 0.001) and S18841 (*F*_(2, 45)_ = 7.77, *P <* 0.01), such that isolates administered the highest dose of S18841 froze significantly more (*P <* 0.01) than vehicle isolates and S18841 restored freezing time in isolates to a level comparable to vehicle group-housed rats. In contrast, freezing time was equivalent in all groups when rats were reintroduced to the context at 48 h post-conditioning, suggesting that extinction was unaltered by housing condition or S18841. However, presentation of the CS 48 h after conditioning increased freezing duration in group-housed more than in isolated rats (Fig. 5g, *F*_(1, 45)_ = 17.45, *P <* 0.001), but this was not reversed by S18841.

## Discussion

Overall, the current study is the first comprehensive comparison of the glycine reuptake inhibitors, sarcosine and ORG24598, the agonists, glycine and D-serine and the partial agonists, S18841 and D-cycloserine on PFC microdialysate amino acid and amine levels and cognitive paradigms in normal rats and a neurodevelopmental model for schizophrenia. Both glycine and GlyT1 inhibitors specifically elevated PFC glycine, while D-serine, and to a lesser extent D-cycloserine, elevated D-serine levels, whereas S18841 had no effect on either amino acid (Table 2). All drugs dose-dependently prevented scopolamine-induced impairment of social recognition in adult rats and over similar dose-ranges attenuated a time-delay-induced reduction of NOR (Table 2). Reversal of NOR deficits by systemic S18841 was prevented by the NMDA receptor antagonist, CPP, and the glycine modulatory site antagonist, L701,324, and the effect of S18841 on NOR replicated by microinjection into the PFC. S18841 also reversed the deficit in NOR and impairment in contextual freezing in a conditioned associative learning task in rats reared in social isolation from weaning (a neurodevelopmental model for schizophrenia). The potential relevance of these findings to cognitive impairments seen in schizophrenia is discussed in detail below.

### Modulation of Extracellular Amino Acids Levels of in PFC

Consistent with extracellular glycine being tightly regulated by GlyT1, and previous work with GlyT1 inhibitors in striatal microdialysates, sarcosine and ORG24598 elevated PFC glycine [30, 51, 67–70] although striatal elevations may be more marked than those in the PFC [37, 70]. Notably, doses of bitopertin achieving high GlyT1 occupancy in humans also elevated CSF glycine suggesting translational pertinence of rat microdialysis studies [71, 72]. Both ORG24598 and sarcosine specifically elevated glycine, without effecting D-serine, other amino acids or acetylcholine, which could contribute to their cognitive effects (Table 1). The only other change was a modest increase in dopamine by sarcosine (Table 1).

These observations are important since, to our knowledge, this is the first comprehensive neurochemical characterisation of sarcosine, despite its extensive use as a specific “GlyT1 inhibitor” and an adjunctive agent in schizophrenia patients [12, 73, 74]. Lack of change in 5-HT is important given concerns about a potential interaction with serotonergic transmission at a clinical dose of 2 g [75] and a transient elevation of rat PFC 5-HT with an extremely high dose (2 g/kg p.o.) of sarcosine [76].

Glycine also elevated dialysate dopamine (and norepinephrine but not 5-HT), the rise being more pronounced than with GlyT1 inhibitors, and completely selective (Table 1) versus other amino acids [51]. It is unclear how glycine elevates dopamine and norepinephrine levels, but this effect is unlikely to involve NMDA receptor recruitment as other glycine site agonists had no effect. One possibility might be a modification of GABA interneuron input to dopaminergic pathways [77]. Increased PFC dopamine release may be advantageous to elevate cognitive and negative symptoms in man [37].

In a mirror image of glycine, D-serine markedly elevated D-serine without affecting glycine [51] or other amines including 5-HT, suggesting that the slight increase in 5-HT in a previous dialysis study [76] resulted from an unselective effect of the much higher dose (2 g/kg) used. Extracellular cortical D-serine levels are dynamically regulated by alanine-serine-cysteine transporters and reflect both neuronal and glial origin [37, 78, 79].

A previous microdialysis study reported that systemic D-cycloserine elevated PFC D-serine levels [80], both in wild-type and serine racemase knockout mice, so it is unlikely to reflect altered synthesis [81]. We confirm herein that D-cycloserine modestly increased PFC dialysis D-serine, again without affecting other amino acids or neurotransmitters (Table 1). As proposed by Horio et al. [81], metabolism of D-cycloserine to D-serine may account for this rise rather than any effect on the glycine site, since neither S18841 (which cannot be transformed into D-serine) nor the antagonist, L701,324, affected D-serine. Moreover, D-serine was unaltered by the other partial agonists. Finally, S18841 exerted no influence at all on extracellular levels of any amino acid (including aspartate and taurine, not shown) or neurotransmitter encouraging us to focus on this pharmacological tool for further studies.

Importantly, demonstration of the highly selective action of sarcosine, ORG24598, glycine (on glycine) and D-serine and D-cycloserine (on D-serine) at doses relevant to those used as adjuncts in schizophrenia clinical trials [10, 17, 26] provides evidence of the likely common PFC neurochemical mechanisms involved in their actions.

### Reversal of Scopolamine-Impaired Social Recognition and Time Delay Deficit in Novel Object Recognition

Scopolamine reproducibly impaired social recognition memory, and this was reversed (in a dose-related manner) by at least two doses of the glycine modulatory site agonists glycine and D-serine, partial agonists D-cycloserine and S18841, and the GlyT1 inhibitors sarcosine and ORG24598. Although the pathological relevance of scopolamine as a cognitive disruptor can be questioned, it has been used as a translational tool to investigate GlyT1 inhibitors in primates and man [82, 83].

All compounds also reduced a time-delay impairment of NOR that was dose-related for glycine, D-serine sarcosine and S18841. These pro-cognitive effects are consistent with previous studies. D-serine and NFPS improved performance in time-delay impairment of social recognition [84]. Spontaneous NOR in mice and place recognition in rats and mice was enhanced by D-cycloserine [85–87]. Supporting the current data that sarcosine and ORG24598 attenuated natural forgetting in NOR performance, glycine reuptake inhibitors NFPS [84] TASP0315003 [88] and sarcosine all improved NOR and NMDA antagonist-impaired social recognition.

### Involvement of the NMDA Receptor and PFC in S18841-Induced NOR Improvement

In this study, reversal of the time-delay-induced impairment of NOR by acute S18841 was prevented by pre-treatment with the glycine site antagonist, L701,324, and the NDMA receptor antagonist, CPP, suggesting that NMDA receptor activation is required for the action of S18841. Consistent with this proposal, D-serine reversed the NOR impairment produced by the NMDA antagonists, MK-801 [87, 89] or PCP [87, 90]. In primates, D-cycloserine improved performance in a variable delayed response task in the MPTP model of Parkinsonism [91] and reversed impairments in a visual delay nonmatching-to-sample task caused by glycine site or NMDA receptor antagonism [92]. Similarly, the partial glycine site agonist, GLYX-13 (Rapastinel), restored a PCP-induced NOR impairment [93]. Both sarcosine and the glycine reuptake inhibitor (R)-(N-[3-(4′-fluorophenyl)-3-(4′-phenylphenoxy)propyl]) reversed an NMDA antagonist-induced impairment of NOR in rats and mice [89, 90] congruent with the proposal that glycine-induced enhancement of NMDA receptor function mediates the reported pro-cognitive effects.

Bilateral microinjection of S18841 into the PFC (but not the striatum) dose-relatedly reversed intertrial delay-impaired NOR. However, the role of the PFC in NOR is complex, such that dopamine D_2_ and D_3_ receptor drugs have opposite effects on performance when injected into the PFC [54, 94]. Furthermore, D-cycloserine injection into the prelimbic cortex reversed scopolamine-induced amnesia in two olfactory (odour discrimination and food preference) tasks [95], suggesting that the cortex may be a target for the observed reversal of muscarinic attenuation of social recognition reported herein. Of note, serine racemase knockout (SR−/−) mice develop neurons with reduced complexity, length and spine density of apical dendrites in the medial PFC and show impaired NOR [96].

### Restoration of Cognitive Performance but Not of Locomotor Activity by S18841 in Isolation-Reared Rats

Rats reared in isolation took longer to habituate to a novel environment, but this hyperactivity, thought to reflect a mesolimbic hyperdopaminergic state analogous to that in psychosis [97], was unaffected by S18841. Current antipsychotics reverse the isolation-induced hyperactivity, at least in part, through antagonism of dopamine D_2_ receptors [46, 49, 98]. Glycine site ligands do not possess D_2_ affinity but may exert antipsychotic properties by a contrasting mechanism or enhance the clinical actions of antipsychotic drugs (although they have never actually been tested alone in patients).

Consistent with previous studies [44–47], isolates showed a marked NOR deficit that was reversed by S18841, suggesting that glycine site agonists can reduce premature forgetting in this neurodevelopmental model of schizophrenia. Only one study has examined the effect of a glycine modulatory compound in isolation-reared rats. D-Cycloserine reversed a deficit in retention in a visual discrimination task [99]. In the present CFR task, social isolation reduced contextual freezing 24 h post-conditioning and when the cue was presented 48 h post-training, consistent with impaired associative memory reported previously [45, 46, 48]. S18841 given prior to conditioning attenuated the isolation-induced reduction in contextual freezing. Interestingly, the contextual memory phase of this task in rats is NMDA receptor dependent, being attenuated by MK-801 [66], consistent with the current hypothesised mechanism of S18841.

### General Discussion and Broader-Clinical Relevance of the Present Observations

As a note of caution both social recognition and NOR tasks rely on spontaneous preference for novelty and their predictive validity to cognitive impairment in schizophrenia requires further confirmation [39, 100, 101]. For instance, drugs with different pharmacological mechanisms, such as α7-nicotinic agonists and 5-HT_6_ receptor antagonists produced much smaller improvements in cognition in schizophrenia clinical trials than would have been predicted from preclinical data, even in studies using disease models rather than normal animals. Nonetheless, in our hands, selective dopamine D_2_ receptor antagonists impair social novelty discrimination and NOR [54] consistent with the lack of effect or worsening of cognition seen with current antipsychotics in patients with schizophrenia, while the dopamine partial agonist cariprazine reversed the isolation-rearing induced impairment in NOR [49].

Taken together, this study provides extensive, consistent complimentary and neurochemical evidence that pharmacological modulation of glycine site activity on NMDA receptors can improve cognitive dysfunction in rats, but similar robust positive observations have not been reported in schizophrenia trials using glycine site agonists [17, 26]. Poor translation may result from desensitisation of the glycine site as seen with repeated D-cycloserine [102], producing NMDA receptor internalisation [103] which might not occur with acute administration used in most animal studies. *Second*, the glycine full and partial agonists appear to have differential affinity for the different NMDA receptor subunit compositions found at pre- and post-synaptic sites in the cortex [104] that could affect their relative clinical effectiveness. *Thirdly*, the ability of glycine site agonists to facilitate symptom improvement may be impeded by antipsychotics with muscarinic antagonism, such as clozapine and other atypicals, by opposing any pro-cognitive actions [94]. Interestingly, D-serine, D-cycloserine and sodium benzoate all improve haloperidol-induced bradykinesia in the rat by elevating nigrostriatal dopamine release, suggesting that they may also reduce antipsychotic-induced extrapyramidal side effects [105]. Furthermore, they might be useful in the prodromal phase to alleviate pre-diagnostic symptoms and, by improving social cognition and social integration, decrease risk of transition to psychosis [27–29, 37].

The present observations provide a framework to examine other agents, including novel glycine site agonists, like Rapastinel [93] and NSX-2925 [14], the uptake inhibitors TASP0315003 [88] and bitopertin [35, 36], D-amino acid oxidases inhibitors like sodium benzoate [22, 106], and inhibitors of kynurenine aminotransferase II all of which modify PFC glutamatergic signalling [107, 108]. Furthermore, it would be interesting to study agents acting at the neuronal alanine-serine-cysteine-1 transporter which is implicated in the control of extracellular D-serine and glycine [8, 109]. Finally, one might compare the actions of BMS-466442, which suppresses D-serine uptake [110], with those of LuAE00527 which prevents D-serine release [79].

Although most research focusses on these agents enhancing cognition in psychiatric disorders [5, 111], current findings are pertinent to disorders, like Alzheimer’s disease [112, 113], where plasma D-amino acid oxidase levels correlate with cognitive decline [114] but other work suggests that elevated brain D-serine occurs in Alzheimer’s [112]. Furthermore, akin to the fast antidepressant actions of ketamine, glycine site partial agonists like Rapastinel may exert antidepressant effects via enhanced mTOR and BDNF signalling [1, 3]. Intriguingly, the major metabolites of ketamine are D-serine racemase inhibitors [1–3], providing a direct link to the present work. In addition, D-cycloserine and other glycine site ligands may improve social cognition in ASDs and genetically related Rett syndrome [115, 116]. Other indications under consideration include drug abuse [117], amyotrophic lateral sclerosis [22], chronic pain [118] and facilitation of psychosocial-cognitive behavioural therapies for anxiety disorders [119].

## Electronic Supplementary Material

ESM 1 (PDF 137 kb)
