# Supplementary material for: Comparative Pro-cognitive and Neurochemical Profiles of Glycine Modulatory Site Agonists and Glycine Reuptake Inhibitors in the Rat: Potential Relevance to Cognitive Dysfunction and Its Management
Source: Mol Neurobiol. 2020 Jan 20;57(5):2144–66. doi: 10.1007/s12035-020-01875-9 (PMC7170834; doi:10.1007/s12035-020-01875-9)
Supplement: Supplementary file 1 — (PDF 137 kb) [file 12035_2020_1875_MOESM1_ESM.pdf]

Supplementary Information for:

Comparative pro-cognitive and neurochemical profiles of glycine modulatory site agonists and glycine reuptake inhibitors in the rat: potential relevance to cognitive dysfunction and its management

Fone[1*] Kevin C.F., Watson[1] David J.G., Billiras[2] Rodolphe I., Sicard[2] D.I., Dekeyne[2] Anne, Rivet[2] Jean-Michel, Gobert[2] Alain & Millan[2*] Mark J.

[1]School of Life Sciences, Queen's Medical Centre, The University of Nottingham, Nottingham, UK, NG7 2UH
[2]Centre for Therapeutic Innovation-CNS, Institut de Recherche Servier, Centre de Recherches de Croissy, 125 Chemin de Ronde 78290 Croissy-sur-Seine, France

*Corresponding authors and equal contribution

E-mail: kevin.fone@nottingham.ac.uk
E-mail: mark.millan@servier.com

Fig. S1.

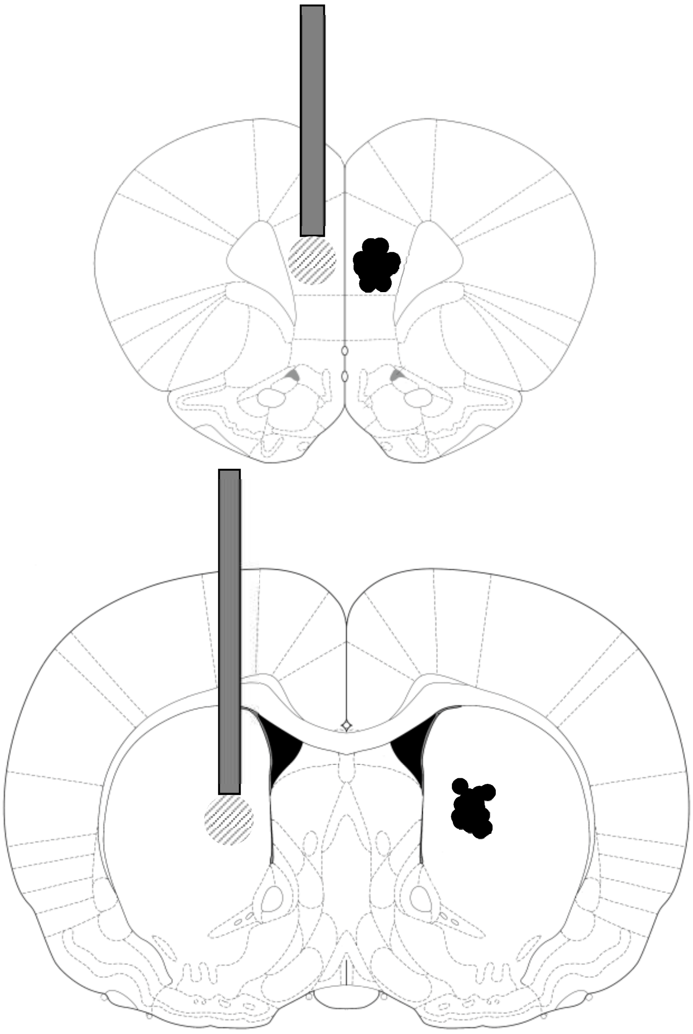

Fig. S1. Confirmation of the position of injection sites (right side) in the rat prefrontal cortex PFC (upper diagram, + 3.2mm from Bregma) and striatum (lower diagram, + 0.48mm from Bregma) from group-housed rats used for dose-response behavioral studies with the micro-injection of S18841 in novel object discrimination shown on coronal sections taken from Paxinos and Watson (1997). Left hand side shows a schematic representation of the injector.

Fig. S2.

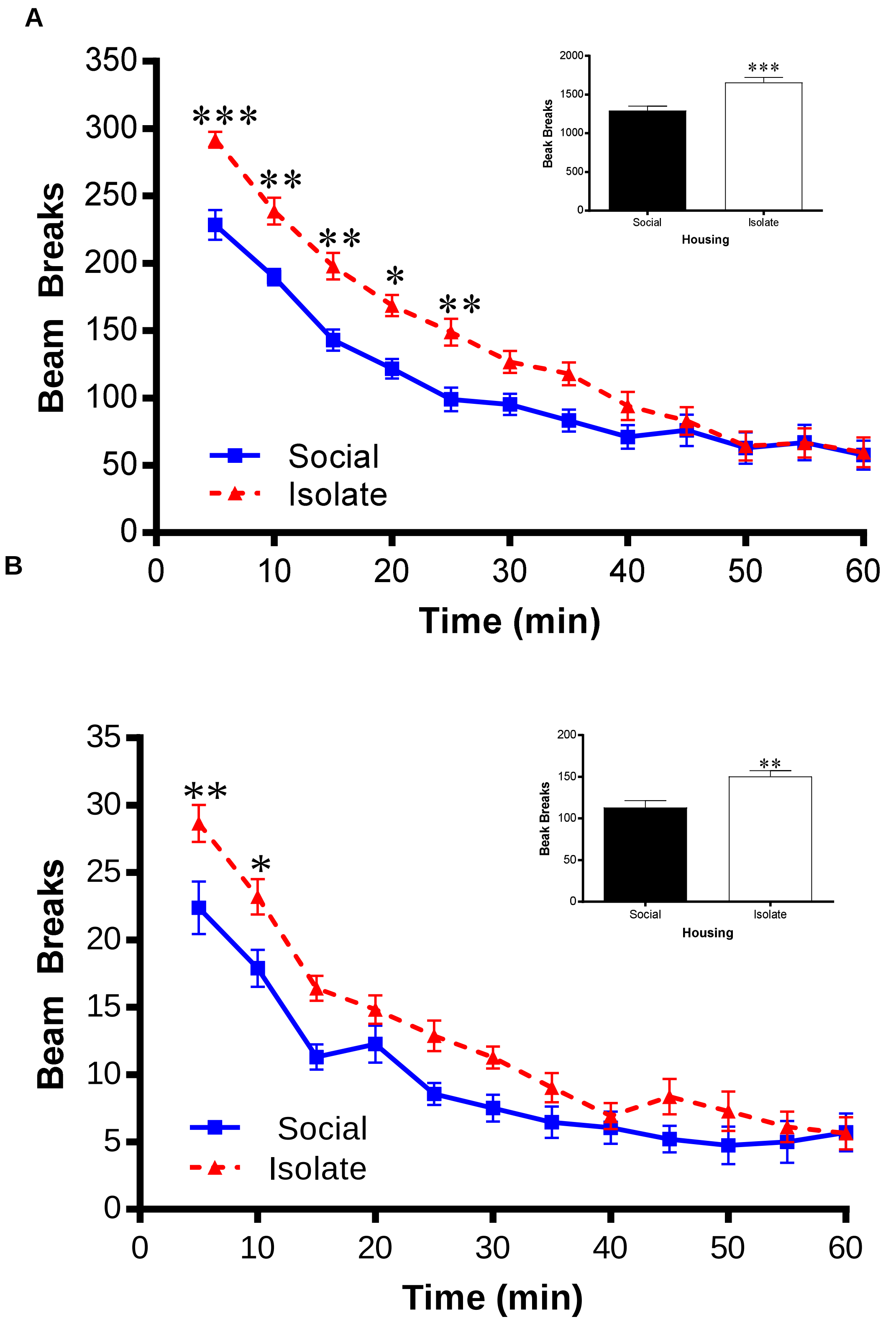

Fig. S2. Social isolation from weaning of male Lister hooded rats caused hyperlocomotor activity (**A**) and an increase in the number of rears (**B**) compared with that observed in

littermates housed in small social groups (n=4) from weaning when they were place in a novel arena (for 1h) 5 weeks after weaning from their dam. Main graph shows the time course of activity counts (mean±SEM, infrared beam breaks) cumulated in consecutive 5min epochs and (inset) the total activity over 1h recorded in group-housed (Social n=20) and individually housed (Isolate n=30) rats to confirm the development of the isolation syndrome prior to performing drug reversal in subsequent weeks. In **A**: RM-ANOVA showed a significant time x housing interaction ($P<0.001$) and a significant main effect of housing (ANOVA, <0.001) * $P<0.05$, ** $P<0.01$, *** $P<0.001$ from the social rats at that time point, Bonferroni *post hoc*. In **B**: RM-ANOVA showed a significant main effects of time ($P<0.001$) and housing ($P<0.01$), * $P<0.05$, ** $P<0.01$. Inset: There was a significant main effect of housing (ANOVA, $P<0.01$) on rears. Isolation reared rats showed the expected hyperactive syndrome compared to group-housed controls.
